# Diagnostic capabilities and monitoring the effectiveness of osteoporosis therapy using radiofrequency echographic multispectrometry in comparison with dual-energy x-ray absorptiometry

**DOI:** 10.3389/fendo.2026.1889333

**Published:** 2026-10-16

**Authors:** Natalia Victorovna Sharashkina, Dmitrii Vladimirovich Rodionov, Nadezhda Konstantinovna Runikhina, Olga Nicolaevna Tkacheva

**Affiliations:** Russian Gerontology Clinical Research Centre, Pirogov Russian National Research Medical University, Moscow, Russia

**Keywords:** antiresorptive therapy, bone imaging, dual-energy x-ray absorptiometry, osteoporosis, radiofrequency echographic multispectrometry

## Abstract

**Purpose:**

This study assessed the potential of the radiofrequency echographic multispectrometry (REMS) for diagnosing and monitoring of osteoporosis over a 12-month period in comparison to the most commonly used diagnostic standard – dual-energy x-ray absorptiometry (DXA) in the Russian population.

**Materials and methods:**

25 women aged from 49 to 88 years were observed with both imaging methods. Outcomes included lumbar spine and femoral neck bone mineral density (BMD) for each method during two visits.

**Results:**

REMS showed moderate diagnostic performance in the present sample. The diagnostic agreement for osteoporosis between DXA and REMS for the two sites was 64% (k = 0.28). Sensitivity and specificity of REMS were 0.63 (0.33 - 0.94 in 0.95 CI) and 0.64 (0.3895 - 0.8894 in 0.95 CI) respectively. However, the comparability of BMD data varied depending on the bone region. Correlation of T-scores for two bone regions between REMS and DXA based on the first visit were 0.65 (P<0.001) and 0.35 (P = 0.089) for lumbar spine and femoral neck respectively. Moreover, Bland–Altman analysis showed poor agreement, with wide agreement limits. Also, collected data revealed a weak correlation for measurements of the second visit within the study, which became statistically insignificant after initiation of antiresorptive therapy. Correlations were 0.47 (P = 0.022) and 0.22 (P = 0.307) for the same two bone regions.

**Conclusion:**

REMS demonstrated statistically significant diagnostic value in the diagnosis of osteoporosis, but the comparability and agreement of BMD data with DXA in the sample was near low and varied widely depending on the assessed bone region and decreased even more during active antiresorptive therapy, which may be due to both the limitations of this study and the characteristics of the method. The present results are not sufficiently precise or statistically robust to validate the method for BMD monitoring unequivocally in Russia, but they provide a basis for further research. A follow-up study in a larger sample is required and is already underway to better substantiate these findings in the Russian population.

## 1 Introduction

Osteoporosis is a systemic metabolic bone disease that leads to deterioration of bone density and it’s micro- and macroarchitecture. It is strongly associated with aging and age-related skeletal changes and remains a major problem in older adults (1), becoming one of the main causes of fractures in old age. Furthermore, secondary osteoporosis often develops as a complication of other common conditions in elderly patients, such as type 2 diabetes mellitus (2), glucocorticoid usage (3), chronic kidney disease (4), and others. In Russia, among individuals aged 50 and older, osteoporosis is diagnosed in 34% of women and 27% of men (5). The high prevalence of osteoporotic fractures after age 50 represents a major public health threat, even compared to dyslipidemia and hypertension (6).

At the same time, the approach to osteoporosis has changed significantly over the decades of its study: it has evolved from a natural age-related alteration to a fully-fledged disease with diagnostic criteria and treatment methods. According to current WHO criteria and relevant at the time of writing clinical guidelines of the Russian Ministry of Health the diagnosis of osteoporosis in adults is established in three cases:

1. The presence of pathological (i.e. low-energy) fractures of large bones of the skeleton, providing exclusion of other skeletal diseases;
2. The presence of a high individual 10-year probability of major pathological fractures according to the FRAX fracture risk assessment tool;
3. A decrease in bone mineral density (BMD) of 2.5 or more standard deviations (SD) - T-score in the femoral neck and/or the entire proximal femur and/or lumbar vertebrae (L1-L4, L2-L4), measured by dual-energy X-ray densitometry (DXA) (7).

Based on these criteria, the diagnosis of osteoporosis is mainly based on the measurement of BMD as the amount of bone mass per unit area or volume (areal BMD, measured in grams per square centimeter [g/cm^2^], or volumetric BMD, measured in grams per cubic centimeter [g/cm^3^], respectively). The following imaging methods are currently available:

1. Classical radiographic analysis
2. Dual-energy X-ray absorptiometry (DXA)
3. Quantitative computed tomography (CT)
4. High-resolution peripheral quantitative CT (HR-pQCT)
5. Magnetic resonance imaging (MRI)
6. Radiofrequency echographic multispectrometry (REMS) (8)

Among the many imaging methods currently available for assessing BMD, dual-energy X-ray absorptiometry (DXA), based on bone X-ray absorptiometry, is the current clinical standard. A detector measures the differential attenuation of two energy X-rays passing through bone and tissue, to calculate BMD or body composition. Diagnosis of osteoporosis, or osteopenia, is made by comparing the individual results with reference values of a young healthy population expressed as the standard deviation (SD) difference from the mean of the reference population (9).

However, the accessibility, the affordability of DXA in low or moderate-income countries like Russia, and the radiation exposure, albeit very low, are driving the search for alternative methods for visualizing BMD and diagnosing osteoporosis. REMS is a relatively new method that enables these tasks by recording the scattering of ultrasound vibrations in tissue. BMD is calculated through an extensive comparison of the specific spectrum of the patient’s bone tissue being examined with a database of reference ultrasound spectral models, and the corresponding T-score and Z-score values are calculated using normative reference databases (10). This method may be a cost-effective, straightforward alternative to DXA, and a number of studies have confirmed its potential utility, especially in primary diagnostics of osteoporosis (11). A recent meta-analysis conducted by R.Y. Liu and E. Liu in 2026 (12) reports a strong correlation between DXA and REMS, but also concludes that further longitudinal studies are needed to assess REMS’s ability to predict fractures in different clinical scenarios, as there are currently not many studies that evaluate the applicability of REMS for dynamic assessment of osteoporosis and risks of fractures. This meta-analysis also mentions substantial heterogeneity, observed across studies, indicating the lack of full universality of correlations across all clinical contexts. Furthermore some of the recent studies suggest that REMS output is strongly based on demographic parameters, rather than an ultrasound-based direct skeletal measurement, especially in some conditions, like perioperative care (13, 14).

Furthermore, at the time of writing, REMS method had been understudied in the Russian population, especially with regard to monitoring in the treatment of osteoporosis.

The aim of this study was to evaluate the REMS method in a population of Russian patients, including geriatric ones, to evaluate the diagnostic accuracy and degree of correlation of REMS data compared with the basic DXA method, to assess sensitivity of detecting changes in BMD in individuals receiving anti-osteoporotic therapy, and to identify the course for further planned research.

## 2 Materials and methods

### 2.1 Data collection

Clinical data were collected in a single-center longitudinal study of female patients who met the following inclusion criteria: age over 18 years and a medically prescribed bone mineral density (BMD) test of the spine and femur using DXA. Patients were recruited from the inpatient and outpatient departments of the Russian Gerontologic Scientific Clinical Center (Pirogov Russian National Research Medical University of Ministry of Health of the Russian Federation), Moscow, Russia. Recruitment period was 6 months (December 2023 to May 2024). Patients were recruited consecutively based on the availability of consent to participate in the study and the possibility of a follow-up visit; the pool of potential participants consisted of all eligible patients in the departments under study. All patients were informed about the nature of the study, data collected, risks and expected benefits; voluntary informed consent was received from all participants.

As part of the study, patients underwent double measurements of BMD parameters using DXA and REMS methods during two visits with a time interval of one year between the initial and repeated measurements.

Before the initial BMD measurements, all patients underwent anthropometry, as well as a medical history and basic clinical data collection, including information on the presence of chronic musculoskeletal diseases, falls and fractures. Body mass index (BMI) was assessed, and a record of current therapy, including anti-osteoporotic medication, was kept. Additional data (laboratory and other anamnestic) was collected from medical records.

During the follow-up visit, anamnestic and general clinical parameters were dynamically assessed, in particular adherence to antiosteoporotic therapy.

Anti-osteoporotic therapy for all participants was standardized according to the current Russian Ministry of Health guidelines for osteoporosis treatment (15). This included the following:

1. Non-pharmacological recommendations, including therapeutic physical training with a set of standard aerobic and anaerobic exercises lasting more than 150 minutes per week, dietary correction with an increase in the content of food rich in protein, calcium, vitamin D, and recommendations for the prevention of falls;
2. Pharmacological treatment: Cholecalciferol, 2,000–7,000 IU depending on baseline serum vitamin D level, Calcium supplements (calcium citrate and calcium carbonate) 500 mg per day, and antiresorptive therapy, which consisted of Zoledronic acid 5 mg intravenously by drip, single injection (1 time per year), or Denosumab 60 mg subcutaneously by injection into the abdominal area 2 times with 6 months interval (2 times per year). The decision on the choice of a specific antiresorptive drug was made by the attending physician in conjunction with the consulting endocrinologist and geriatrician, as required, based on the individual clinical and laboratory data of patients, standardized by clinical guidelines and Instructions for use of specific medicinal products.

All participants had BMD measured at both times at the lumbar spine and femoral neck using DXA (as prescribed) and REMS.

DXA scanning was performed using the GE HealthCare^®^ Lunar Prodigy™ Advance DXA system according to standard clinical procedures. During the spine and femur X-ray densitometry examination, the lumbar region and femur were positioned on the table so that their longitudinal axes were parallel to the vertical edge of the image.

Scanning of the lumbar vertebrae and proximal femur using the REMS method was performed via Echo Station ultrasound machine (EchoLight S.p.a., Italy), equipped with a convex transducer with a nominal frequency of 3.5 MHz for each scan, a final medical report, the corresponding B-mode image sequence, and raw ultrasound data were automatically saved.

EchoStudio software, which is a biomedical software used in conjunction with Echo S/EchoStudio evaluates bone mineral density using the patented REMS densitometry method. EchoStudio enables the evaluation of key diagnostic parameters directly on anatomical structures with an increased fracture risk: the lumbar spine and proximal femur. EchoStudio analyzes ultrasound signals and echographic images to calculate diagnostic parameters (BMD, T-score and Z-score) and to assess 10-year fracture risk using EchoLight diagnostic algorithms and non-ionizing REMS method.

For each vertebra or femur scan, the operator set the transducer focus and scanning depth to place the target bony interface (i.e., the lumbar vertebral surface or femoral neck profile) within the focal zone of the ultrasound beam to visualize the object approximately in the middle of the B-mode image and at least 3 cm from the inferior edge.

Specifically, when scanning the lumbar spine (L1 - L4), the patient is placed supine. After applying ultrasound gel to the patient’s skin, the operator positions the ultrasound transducer transabdominally under the sternum to center the L1 vertebral profile in the middle of the reconstructed B-mode image. The transducer is then moved distally through the L2, L3, and L4 vertebrae, following on-screen instructions and audio signals provided by the device’s software as visual feedback. For optimal scanning depth and focal positioning for the lumbar region, the vertebral bone junction should appear in the middle of the ultrasound image, parallel to and directly below the focal line. Scanning one region takes an average of 80–90 seconds, and the progress bar turns green and displays the scan progress until completion. After the scan is complete, EchoStudio analyzes the acquired data and displays the corresponding medical report.

During femoral neck scanning, the patient lies supine. The operator positions the ultrasound transducer parallel to the femoral head-neck axis to visualize the profile of the proximal femur, including the boundaries of the femoral head, neck, and greater trochanter, within the B-mode image field of view, aiming to visualize a longitudinal section of the head and trochanter.

The examination was performed by specially selected researchers who had undergone preliminary training in the use of the equipment and the examination methodology. After the scan EchoStudio analyzes the results and generates a medical report.

The medical report obtained from the ultrasound scan contains patient data and a graph of diagnostic parameters (BMD, T-score and Z-score) calculated for the corresponding scanned area, taking into account age, gender, and other variables. The graphic image of results is colored according to the standard color pattern used by FRAX.

The report also includes numerical values for the parameters (BMD, T-score, and Z-score). The patient’s diagnostic classification (normal, osteopenia, osteoporosis) is assessed based on the T-score according to the WHO definition.

### 2.2 Statistical analysis

R ver 4.5.0 2025-04–11 ucrt (^©^The R Foundation) with packages, Rstudio 2026.01.2 Build 418 software (^©^ Posit Software, PBC) was used for statistical analysis.

The analysis included an assessment of the general statistical characteristics of the sample. The normality of the data distribution was assessed using the Shapiro-Wilk test. Quantitative characteristics were expressed as mean values with standard deviations and medians with ranges; categorical variables are shown as frequencies.

A comprehensive analysis of medical data was performed for the lumbar spine and femoral neck for both DXA and REMS. Using T-score values obtained from DXA and REMS measurements in each anatomical region, each patient was classified according to the presence of osteoporosis for each of the performed tests: with a T-score ≤-2.5 as osteoporosis, with a T-score>-2.5 – without. In addition, stratification by osteoporosis and osteopenia was assessed according to generally accepted criteria for each region separately.

To analyze differences between the groups with and without osteoporosis, an independent two-sample t-test (for normally distributed quantitative variables), the Mann-Whitney test (for other quantitative variables), and the chi-square test (for categorical variables) were used. The chi-square test was also used to determine the difference in diagnostic accuracy between the presence of osteoporosis according to REMS and the “gold standard” for osteoporosis determination, DXA. The level of statistical significance was set at <0.05. Additionally, a Kernel probability density estimation (KDE) was performed.

The diagnostic accuracy of REMS was then assessed relative to DXA as a reference, expressed as a percentage, and the agreement between the two methods was assessed by the same criterion by calculating the corresponding Cohen’s kappa coefficient (k). Sensitivity and specificity were then determined for distinguishing between patients with and without osteoporosis. Sensitivity was calculated as the proportion of subjects with osteoporosis based on the reference DXA who were identified as positive by REMS. Specificity was calculated as the proportion of subjects without osteoporosis based on the reference DXA who were identified as negative by REMS. The positive predictive value (PPV) was calculated as the probability of actually having osteoporosis when REMS detected it, while the negative predictive value (NPV) was calculated as the probability of actually not having osteoporosis when REMS did not detect it. The confidence interval was set within 0.95, and the calculation of confidence intervals was carried out using the “bootstrap” method.

Correlation analysis of the data of measurements of 2 bone regions via REMS and DXA was carried out using the Pearson correlation coefficient (due to the normal distribution of data), the confidence interval was set within 0.95, the significance level value (p) was calculated.

Finally, the agreement between T-score values measured by REMS and DXA was directly assessed by constructing scatter plots and using the Bland-Altman method for each pair of data.

The calculation of correlation and agreement of data for measurements obtained during the follow-up visit was performed similarly to that described above for the initial visit.

## 3 Results

A total of 40 patients were examined. Based on the screening results for inclusion/exclusion criteria, 32 patients with clinical indications for bone densitometry were included in the study. They underwent both initial DXA and REMS assessments from December 2023 to May 2024. The observation period for each patient was at least 1 year. During prospective observation, 7 patients withdrew from the study for the following reasons: 1 patient – loss of contact (fate unknown), 3 patients – refusal of a follow-up visit, 3 patients – refusal of further follow-up.

Thus, among the included patients who completed the study at both visits, 25 women aged 49 to 88 years were observed. BMI ranged from 22 to 41.9 kg/m^2^, with a mean value of 27.57 kg/m^2^ (1–3 quartiles - 24.4-29.85 kg/m^2^).

Antiresorptive therapy, besides standard universal treatment, was the following: 14 patients received Zoledronic acid, while the remaining 11 received Denosumab. All participants had high adherence to therapy with no significant deviations in the implementation of appointments.

### 3.1 First visit

The baseline data collected during the first visit were assessed and presented below as mean values and comparisons between groups according to the presence of osteoporosis.

The mean lumbar vertebrae BMD T-score values at the first visit were -1.89 (± 1.32) according to DXA and -2.33 (± 0.95) according to REMS. The mean femoral neck BMD T-score values at the first visit were -1.95 (± 0.81) according to DXA and -2.2 (± 0.92) according to REMS.

The distribution of data on the presence of osteoporosis and osteopenia depending on the examined area of bone tissue at the first visit is presented in **Table 1**. The main statistical parameters of the studied sample are presented in **Tables 2**, **3**.

**Table 1:** Distribution of the sample by the presence of osteoporosis and osteopenia at two bone regions at the first visit.

Parameter	DXA of the L1–4 spine section	DXA of the femoral neck	REMS of the L1–4 spine section	REMS of the femoral neck
Normal	2	7	6	3
Osteopenia	12	13	9	12
Osteoporosis	11	5	10	10

**Table 2:** Mean values (mean and standard deviation/median with min-max interval) of the main numerical parameters studied in the sample.

Parameter	Mean (± sd)	Median (min-max)
Age at the time of the study	72.33 (± 10.53)	73 (49-88)
Body weight	67.09 (± 9.26)	65 (52-84)
Height	159 (± 14.11)	162 (110-179)
BMI	27.57 (± 4.51)	27.8 (22-41.9)
Menopause (age, years)	50.53 (± 3.94)	50 (43-55)
Vit. D level (ng/ml)	35.43 (± 18.17)	41.96 (2.37-57.8)
Calcium serum level (mmol/L)	2.36 (± 0.76)	2.36 (0.86-3.68)
T -score L1-L4 lumbar spine DXA (1st visit)	-1.89 (± 1.32)	-2.3 (-4.4-1)
T -score of femoral neck DXA (1st visit)	-1.95 (± 0.81)	-2.1 (-3.4--0.5)
TBS L1-L4 DXA	1.26 (± 0.14)	1.25 (1-1.52)
T -score L1-L4 lumbar region REMS (1st visit)	-2.33 (± 0.95)	-2.3 (-3.8-0.6)
T -score femoral neck REMS (1st visit)	-2.2 (± 0.92)	-2.1 (-3.7-0.4)

**Table 3:** Frequencies (given as absolute values and percentages) of the main categorical parameters in studied sample.

Parameter	Frequencies		
Gender: 0 - F, 1 - M	0: 2 5 (100%)	1:0	
Previous fractures: 1 - yes, 0 - no, 3 - multiple	0: 19 (76%)	1:6 (24%)	3:0
Hip fracture in parents: 0 - no, 1 - yes	0:25 (100%)	1:0	
Current smoking: 0 - no, 1 - yes	0:25 (100%)	1:0	
Glucocorticoids: 0 - no, 1 - yes	0:25 (100%)	1:0	
Rheumatoid arthritis: 0 - no, 1 - yes	0:23 (92%)	1:2 (8%)	
Secondary osteoporosis: 0 - no, 1 - yes	0:25 (100%)	1:0	
Alcohol: 0 - no, 1–3 or more units per day	0:25 (100%)	1:0	
Osteoporosis by DXA: 0 – no, 1 – yes	1: 11 (44%)	0:14 (56%)	
Osteoporosis by REMS: 0 – no, 1 – yes	1:12 (48%)	0: 13 (52%)	

The results of the assessment of intergroup differences between patients with and without osteoporosis revealed the following dependent parameters (statistical significance values p are given in parentheses): age (<0.001), height (0.0242), and the presence of previous fractures (0.0031). General data of intergroup comparisons are presented in **Tables 4**, **5**. KDE graphs by groups for age and body mass parameters are presented in **Figures 1**, **2**.

**Table 4:** Results of intergroup comparison of non-categorical parameters between patients with and without osteoporosis at the first visit, p – statistical significance; values of p <0.05 are highlighted in bold.

Parameter	*p*
Age at the time of the study	**<0.001**
Body weight	0.3926
Vit. D level (ng/ml)	0.1629
Calcium serum level (mmol/L)	0.1409
T -score L 1- L 4 lumbar region REMS (1st visit)	**<0.001**
T -score L1-L4 lumbar region REMS (2nd visit)	**<0.001**
T -score femoral neck REMS (1st visit)	**<0.001**
T -score femoral neck REMS (2nd visit)	**0.0056**
Height	**0.0242**
BMI	0.6437
Menopause (age, years)	0.2469

**Table 5:** Results of intergroup comparison of certain categorical parameters between patients with and without osteoporosis at the first visit, p – statistical significance; values of p <0.05 are highlighted in bold.

Parameter	*p*
Gender: 0 - F, 1 - M	0.84739
Previous fractures: 1 - yes, 0 - no, 3 - multiple.	**0.0031**
Rheumatoid arthritis: 0 - no, 1 - yes	0.13965

**Figure 1:**
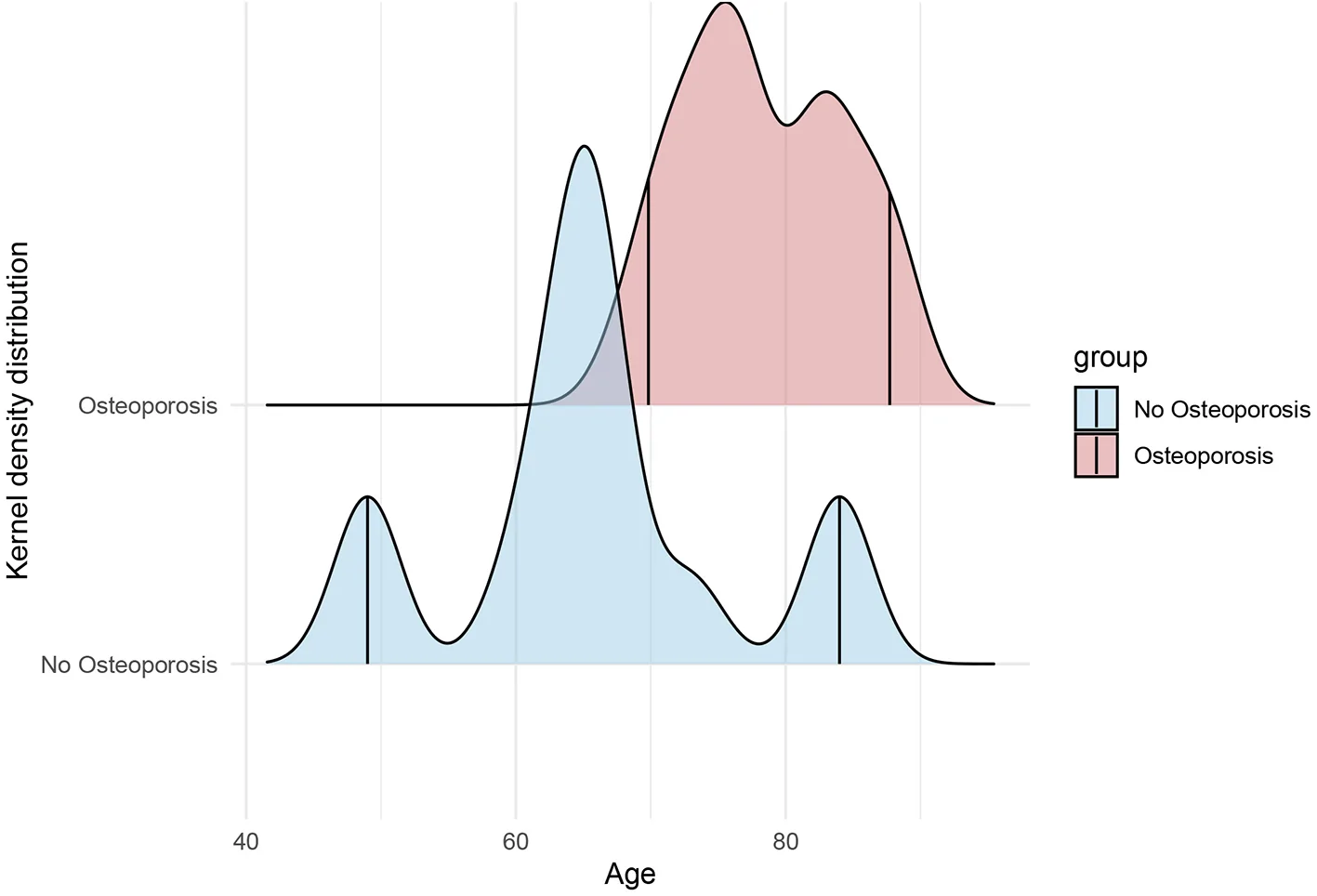
Graph of comparative nuclear density assessment for groups of patients with and without osteoporosis depending on age (in years). Quantiles (0.025, 0.975) shown as vertical cutoffs.

**Figure 2:**
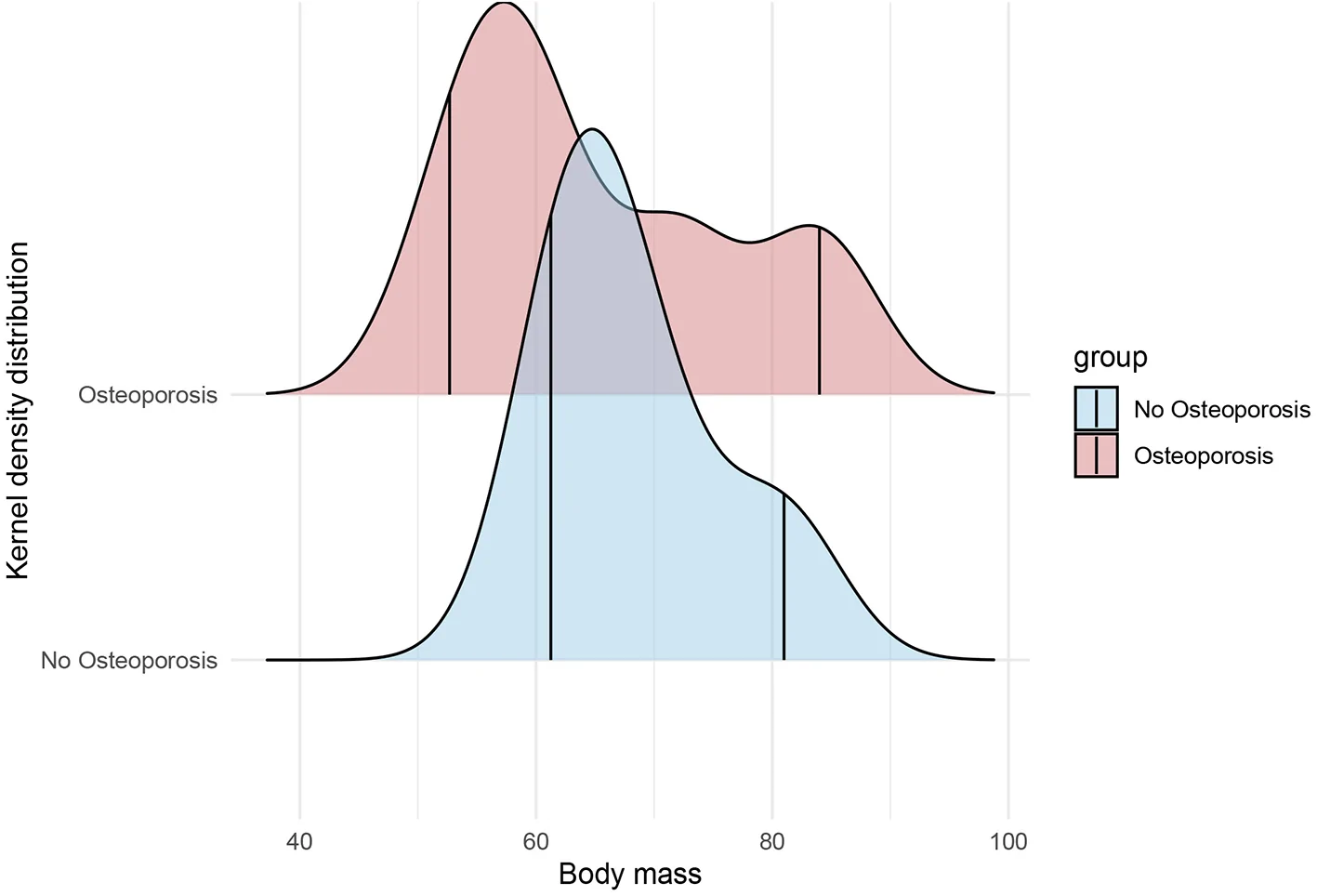
Graph of comparative nuclear density assessment for groups of patients with and without osteoporosis depending on body mass (kg). Quantiles (0.025, 0.975) shown as vertical cutoffs.

Diagnostic agreement for osteoporosis between DXA and REMS across the two bone sites was 64%, with Cohen’s kappa k = 0.28. The diagnostic agreement for osteoporosis and osteopenia for the L1-L4 spine zone data between similar measurements was 52%, and for the femoral neck data – 80%. The sensitivity and specificity values for REMS relative to DXA were the following: Sensitivity -0.6364 (CI 0.33 - 0.94), Specificity - 0.6429 (CI 0.39 - 0.89), Positive predictive value - 0.5833 (CI 0.31 - 0.87), Negative predictive value - 0.6923 (CI 0.42 - 0.96). These data indicate moderate sensitivity, specificity, and predictive performance, but also had modest agreement in actual diagnostic capabilities. The confusion matrix is presented in **Table 6**.

**Table 6:** Confusion matrix of osteoporosis between DXA and REMS during the first assessment; green and red shading highlight true and false prediction, respectively.

Presense / Predition	Sample size = 25	Osteoporosis prediction by REMS	
		Positive prediction	Negative prediction
Actual osteoporosis prevalence by DXA	Positive	7	4
	Negative	5	9

The results of the correlation analysis using the T-score for two bone regions between REMS and DXA based on the first visit data were the following: correlation between REMS lumbar T-score L1-L4 and DXA lumbar T-score L1-L4 was 0.6471 (CI 0.34 - 0.83, *p=*0.0004), correlation between REMS femoral neck T-score and DXA femoral neck T-score was 0.3472 (CI 0 - 0.65, *p=*0.089). Specifically, lumbar spine measurements performed at the first visit showed a moderate positive statistical relationship, whereas BMD data at the femoral neck showed a weak correlation.

Results of agreement assessment via Bland-Altman method showed ambiguous results indicating poor agreement: while bias of T-scores was close to 0 (0.188 for lumbar spine and 0.299 for femoral neck), limits of agreement in 95% interval were wide (-1.543~1.919 for lumbar spine and -1,516~2.116 for femoral neck).

Scatter plots and Bland-Altman plots for REMS and DXA BMD results are shown in **Figures 3**–**6**.

**Figure 3:**
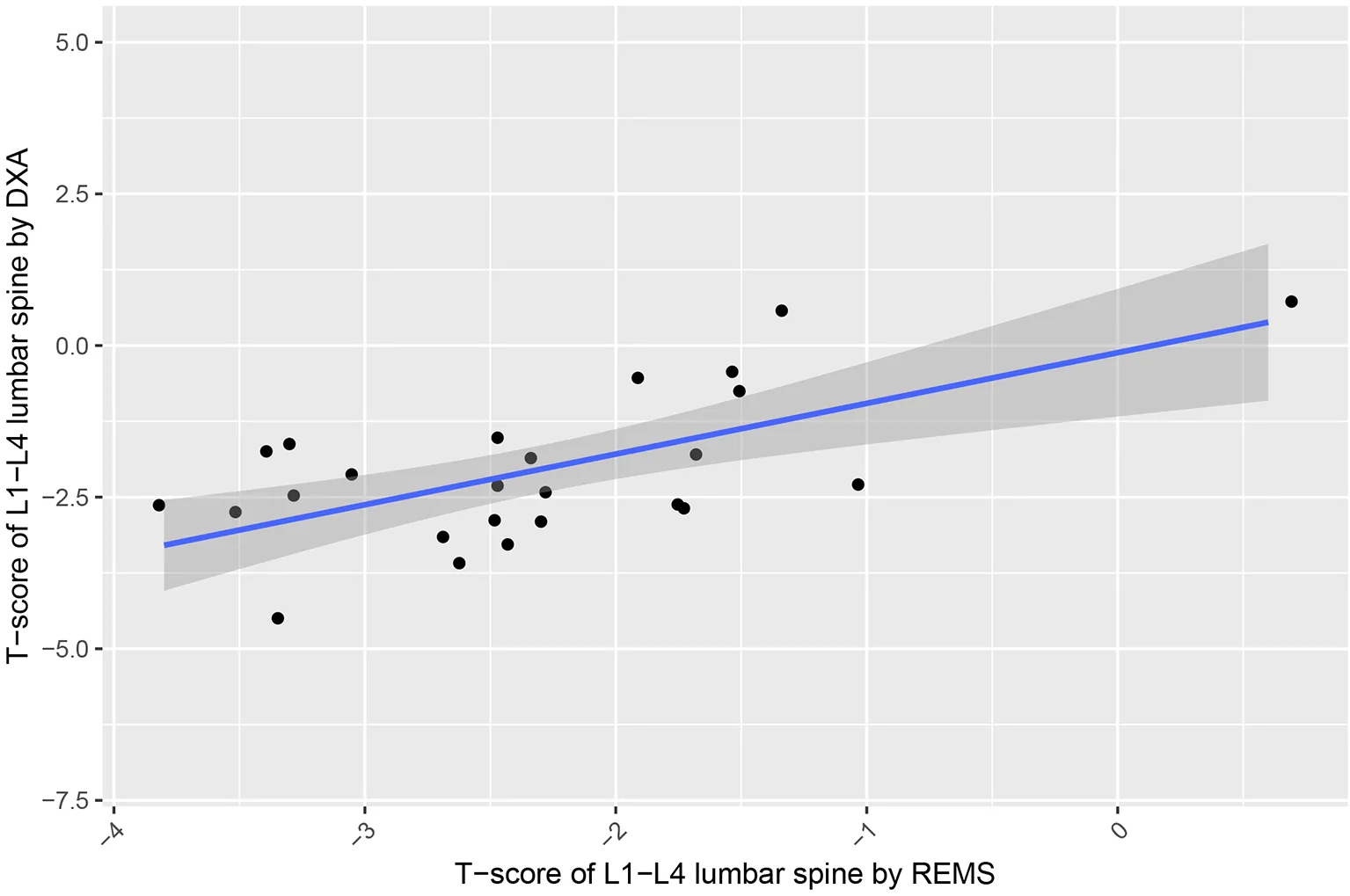
Scatterplot between REMS and DXA lumbar vertebrae BMD T-score data, 1^st^ visit.

**Figure 4:**
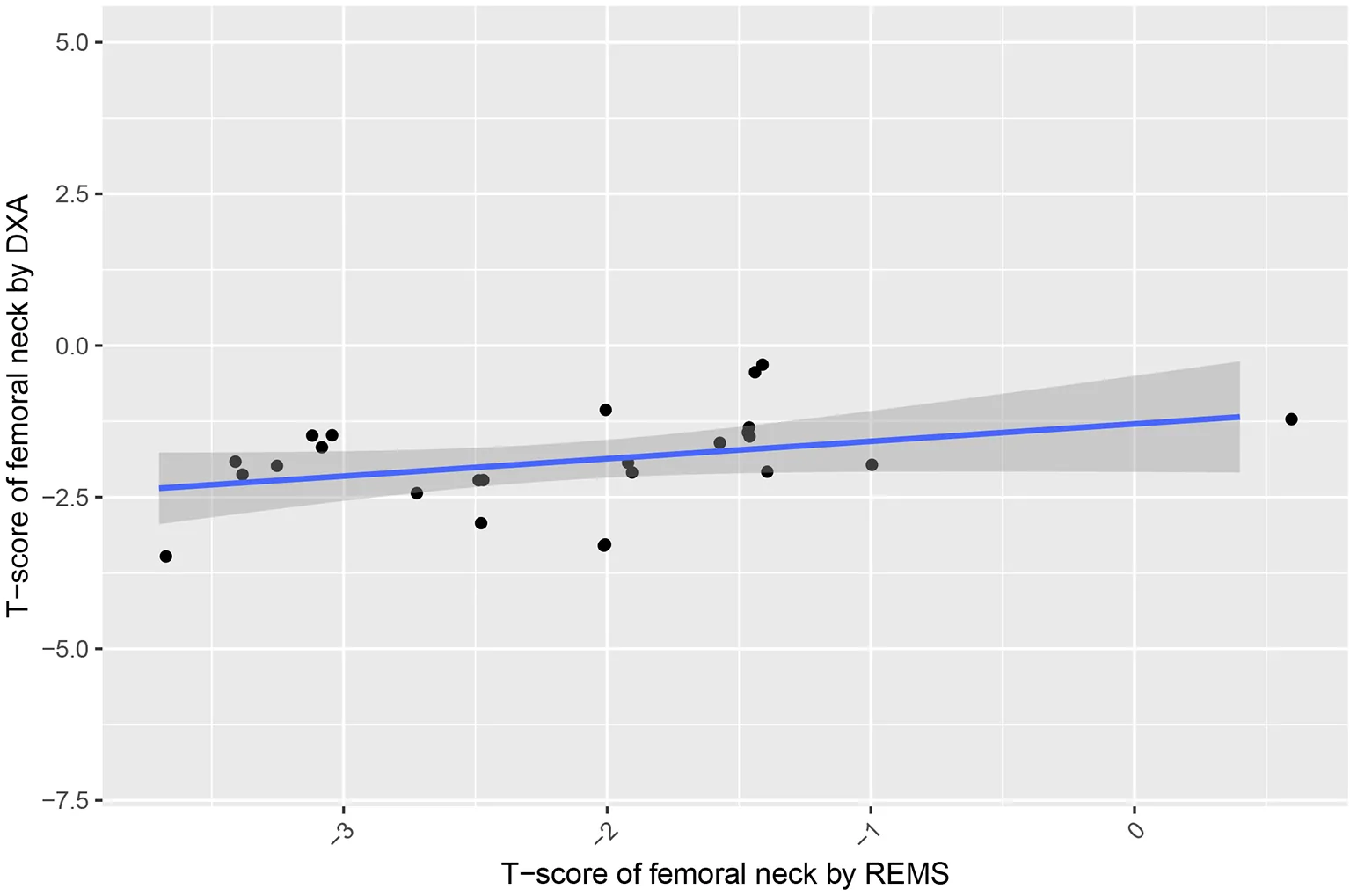
Scatterplot between REMS and DXA femoral neck BMD T-score data, 1^st^ visit.

**Figure 5:**
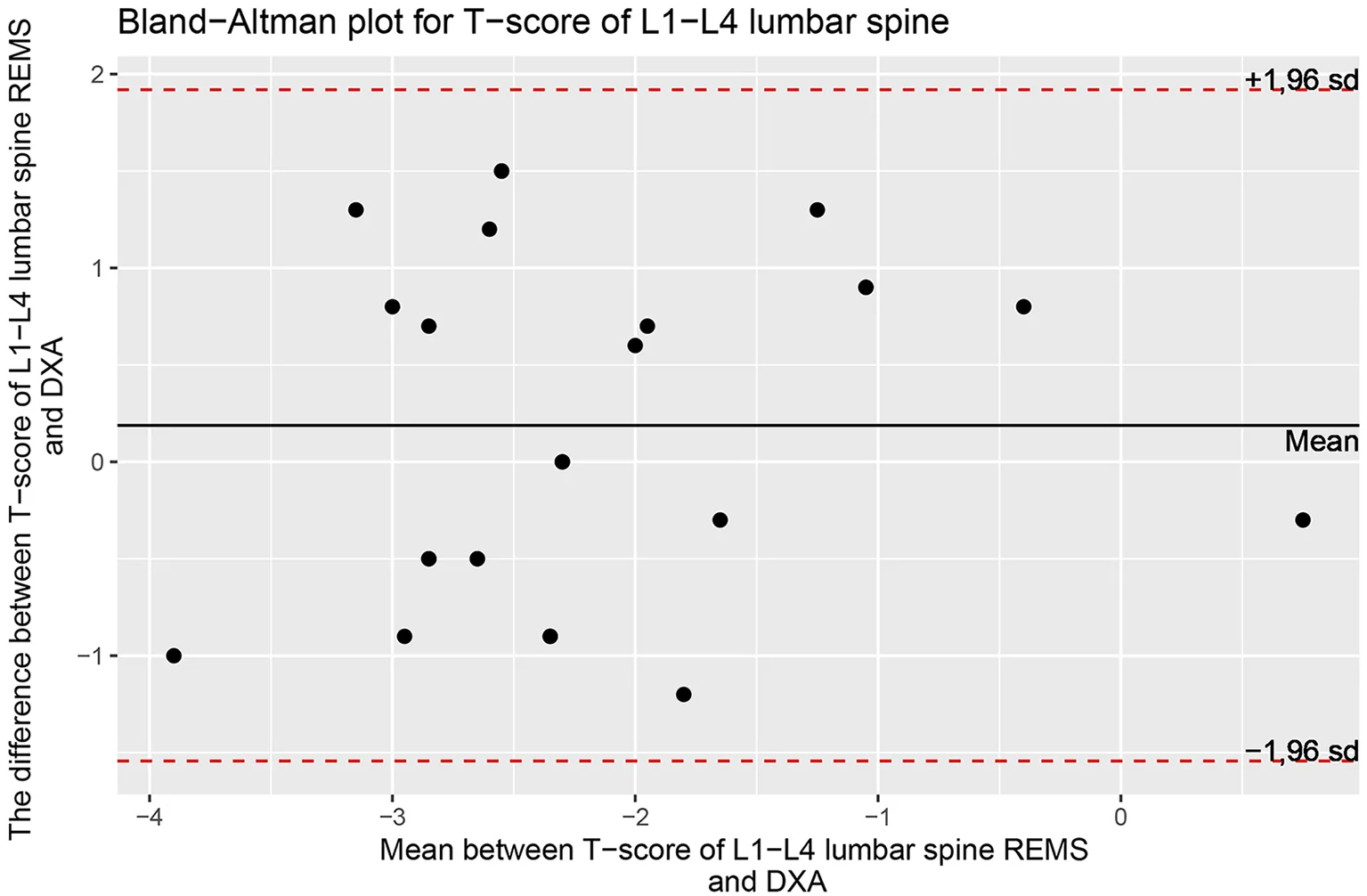
Bland-Altman plot for REMS and DXA BMD results, lumbar vertebrae BMD T-score data, 1st visit.

**Figure 6:**
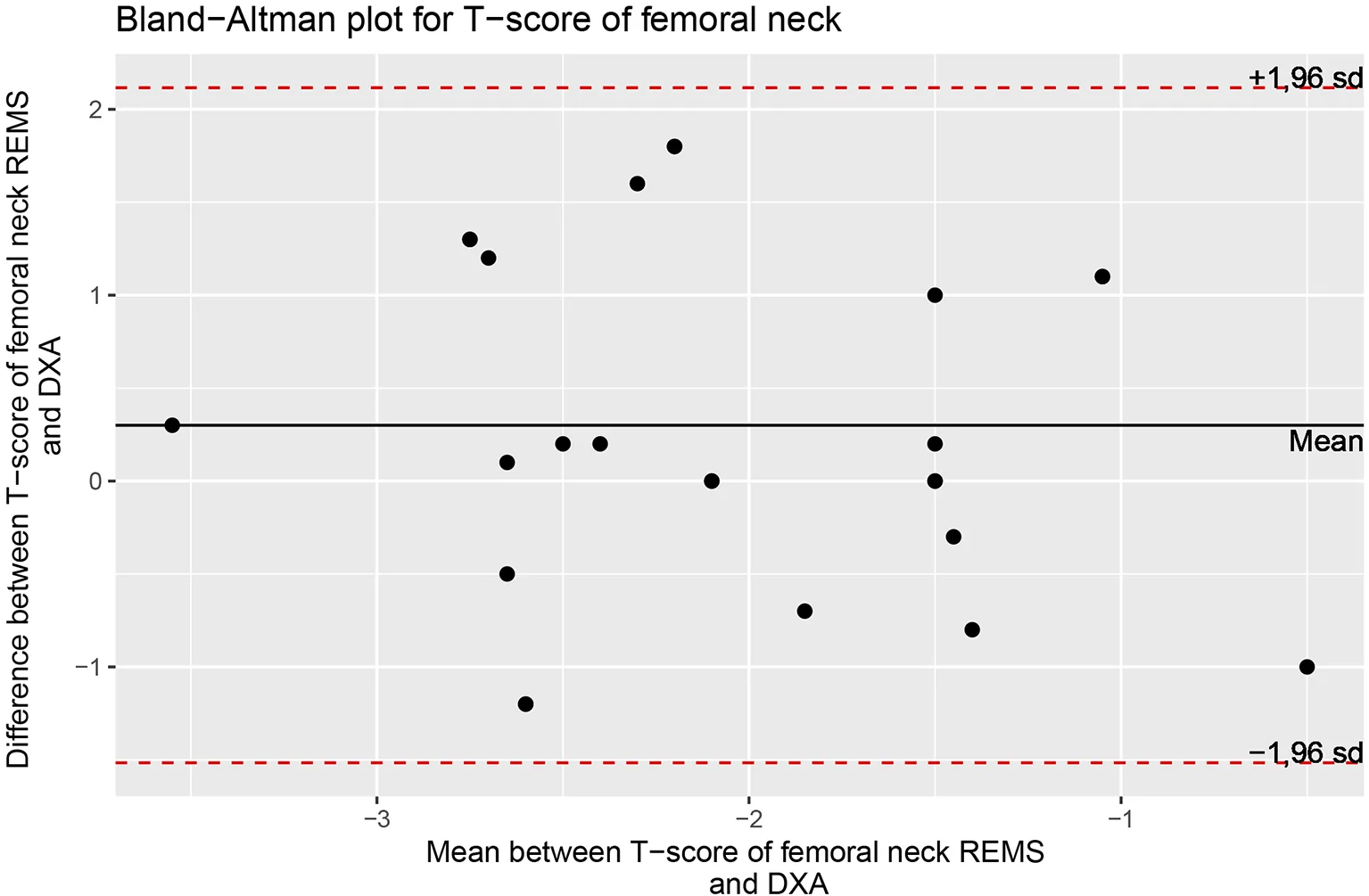
Bland-Altman plot for REMS and DXA BMD results, femoral neck BMD T-score data, 1st visit.

### 3.2 Second visit

The BMD data of patients during the second visit had the following mean values: -1.43 (± 1.2) and -2.21 (± 0.91) for the T-score of BMD of the lumbar vertebrae, -1.96 (± 0.79) and -2.06 (± 0.92) for the T-score of BMD of the femoral neck according to DXA and REMS data, respectively. Results of intergroup comparison of REMS T-score for two bone regions between patients with and without osteoporosis were p<0,001 for L1-L4 lumbar spine section and p=0.0056 for femoral neck. Mean values of the T-score for REMS and DXA for the second visit are shown in the **Table 7**.

**Table 7:** Mean values (mean and standard deviation/median with min-max interval) of the T-score of the examined bone regions at the second visit according to DXA and REMS data.

Parameter	Mean (± sd)	Median (min-max)
T-score L1-L4 lumbar DXA (2nd visit)	-1.43 (± 1.2)	-1.35 (-4-0.8)
T-score femoral neck DXA (2nd visit)	-1.96 (± 0.79)	-2 (-3.4--0.8)
T-score L1-L4 lumbar REMS (2nd visit)	-2.21 (± 0.91)	-2.1 (-4.2-0.1)
T-score femoral neck REMS (2nd visit)	-2.06 (± 0.92)	-2.2 (-4.7-0.4)

Assessment of correlations of data between REMS and DXA in two bone regions based on the second visit resulted in the following values: correlation between REMS lumbar T-score L1-L4 and DXA lumbar T-score L1-L4 was 0.4651 (CI 0.08 - 0.73, *p=*0.022), correlation between REMS femoral neck T-score and DXA femoral neck T-score was 0.2176 (CI 0 - 0.57, *p=*0.3). These data revealed a weak correlation within the study, which became statistically insignificant for the femoral neck T-score after initiation of antiresorptive therapy.

## 4 Discussion

In the presented results, the REMS method demonstrated moderate sensitivity, specificity, and predictive value (both positive and negative). However, the diagnostic performance was moderate: although actual diagnostic accuracy was fairly high, diagnostic agreement as measured by Cohen’s kappa was modest. The correlation between the REMS study and the standard diagnostic procedure for osteoporosis (DXA) was also moderate for lumbar BMD and insignificant for femoral neck BMD. Also Bland–Altman analysis showed poor agreement with wide limits of agreement.

Moreover, according to the data collected during the second visit, it is clear that for both bone regions, a significant decrease in the correlation of BMD data was determined after the start of therapy, especially for the femoral neck.

Many multicenter studies with large numbers of patients have demonstrated high agreement between REMS and DXA. A Russian data comparability study conducted on 50 patients, published in 2025, also demonstrated a high correlation between REMS and DXA (16). Some studies with smaller numbers of subjects have obtained results similar to this study (11). For example, in the study by Caffarelli C. et al., which included 77 people, the correlation between the data was 0.35 and 0.54 for the lumbar spine and femoral neck, respectively (17). Furthermore, even in the largest studies, the sensitivity and specificity of osteoporosis detection by REMS were similar to those of this study (see, for example, Adami G. et al. (18)).

The structure of the study population, in terms of body weight and composition, as well as the presence of pathological fractures, may have played a role in the obtained results. For example, in a study by Ishizu H. et al., when identifying and analyzing subgroups of patients based on the degree of agreement between REMS and DXA data, significant discrepancies were found among those with elevated BMI. The same study also found that the presence of pathological fractures, type 2 diabetes, and certain other conditions can also significantly impact the agreement between DXA and REMS data (19). The recent meta-analysis has shown, that the strength of the correlation may be substantially weaker in certain settings or populations (12). Furthermore, there are no data from trials of REMS devices with a BMI greater than 40 kg/m^2^.

In a number of studies, to determine the sensitivity and specificity of detecting osteoporosis for diagnostic criteria, a deviation of 0.3 from the cutoff value of T-criteria was defined as acceptable (see, for example, Di Paola M. et al. (20)), which significantly improved the assessment result; however, in Russia, this subanalysis does not make sense, since the criteria established by the Russian Ministry of Health at the time of writing are strict and do not allow deviations.

Another important factor that can significantly influence the results is the possibility of study errors. In the meta-analysis conducted by Icătoiu E. et al. (11), an important criterion for reviewing the literature was the analysis of errors, such as incorrect patient positioning during instrumental assessment, various artifacts, errors in reporting patient data, and others.

The study also revealed a significantly lower agreement between REMS and DXA data at the femoral neck compared to the lumbar spine. These observations are consistent with many similar studies (11). It should also be noted that in the studies with high agreement for lumbar spine data, excluding patients with significant degenerative changes in the lumbar spine from the analysis was crucial, as the described advantage of the technique is the automatic exclusion of bone osteophytes and soft tissue calcifications from the analysis (20). This advantage allows for the possibility of a more accurate diagnosis of bone condition based on REMS data for the lumbar spine, as the results of examination of the proximal femur are currently considered the most reliable data.

An important identified trend in terms of REMS application was a significant decrease in correlation after the initiation of anti-osteoporotic therapy during the follow-up visit. This phenomenon may be due to differences in the physical basis for recording bone mineral density using these methods. Since the follow-up visit was conducted one year after the initial visit, when the effect of anti-osteoporotic therapy may not have had time to significantly alter bone microarchitecture but only alter the amount of hydroxyapatite, this phenomenon requires further investigation, including by examining changes in data consistency over a longer period.

It is also worth noting that some studies have raised concerns about inconsistencies between DXA as conventional “gold standard” and other methods of radiographic of BMD assessment (e.g., CT) (21). This doesn’t diminish the importance of DXA but requires further clarification and consideration, e.g. by introducing other BMD measurement methods in study design.

A significant limitation of the study is its sample size, which, however, could not be increased within the framework of this initial diagnostic, research and administrative capabilities, since the procedure was new in the study center, and significant time and resources were required to establish the workflow, as well as to inform patients about new diagnostic options.

Finally, it is worth noting that all patients included at this stage of the study were female, which undoubtedly limits the general applicability of the results obtained.

While this study evaluated a small number of patients, despite the limitations noted above, the results obtained are sufficient to suggest a potential diagnostic role for REMS, particularly in primary screening. However, the wide limits of data agreement between REMS and DXA identified at the current phase of the study, as well as the loss of agreement in the phase of treatment monitoring does not allow to state the high diagnostic performance of REMS dynamic assessment of osteoporosis, especially based on the current small sample. A clearer assessment of the diagnostic performance and monitoring utility of this method requires a more detailed evaluation in terms of data consistency and agreement relative to the «gold standard». Similar conclusions follow from the Expert Consensus on the Diagnosis of Osteoporosis and Sarcopenia in the Elderly, published in Russia in 2023 (22).

To obtain better results, the extended study of the REMS methodology is planned to be improved in the following areas:

1. clarification of criteria, increase in the number and expansion of the demographic spectrum of subjects studied to increase the power and universality of the results;
2. conducting an analysis of inter-rater and test-retest reliability and reproducibility, taking into account possible researcher errors;
3. accounting and analysis of subgroups studied according to the parameters of the presence of obesity, pathological fractures, diabetes mellitus and some others;
4. conducting separate statistical analysis of sensitivity and specificity for osteoporosis and osteopenia separately;
5. recording and analysis of the fragility index (Fragility Score) obtained by the device software.

The follow-up study is required and planned for implementation.

## Data Availability

The raw data supporting the conclusions of this article will be made available by the authors, without undue reservation.

## References

[1] Salari N Ghasemi H Mohammadi L Behzadi MH Rabieenia E Shohaimi S . The global prevalence of osteoporosis in the world: a comprehensive systematic review and meta-analysis. J Orthop Surg Res. (2021) 16:609. doi: 10.1186/s13018-021-02772-0 34657598 PMC8522202

[2] Sheu A Greenfield JR White CP Center JR . Assessment and treatment of osteoporosis and fractures in type 2 diabetes. Trends Endocrinol Metab. (2022) 33:333–44. doi: 10.1016/j.tem.2022.02.006 35307247

[3] Rahman A Haider MF . A comprehensive review on glucocorticoids induced osteoporosis: a medication caused disease. Steroids. (2024) 207:109440. doi: 10.1016/j.steroids.2024.109440 38754651

[4] Evenepoel P Cunningham J Ferrari S Haarhaus M Javaid MK Lafage-Proust M-H . European consensus statement on the diagnosis and management of osteoporosis in chronic kidney disease stages G4–G5D. Nephrol Dialysis Transplant. (2021) 36:42–59. doi: 10.1093/ndt/gfaa192 33098421

[5] Lesnyak OM . Audit of the state of osteoporosis in eastern europe and central asia 2010. Osteoporosis osteopathy. (2011) 14:3–6. doi: 10.14341/osteo201123-6

[6] Borgström F Karlsson L Ortsäter G Norton N Halbout P Cooper C . Fragility fractures in Europe: burden, management and opportunities. Arch osteoporosis. (2020) 15:59. doi: 10.1007/s11657-020-0706-y 32306163 PMC7166207

[7] Ministry of Health of the Russian Federation . Osteoporosis: ICD 10: M81.0, M81.8: Clinical guidelines (2025). Available online at: https://cr.minzdrav.gov.ru/view-cr/87_5 (Accessed May 23, 2026).

[8] Poorvitha HR Chandrakala BM . A review of osteoporosis detection based on image texture of inner trabecular bone microarchitecture using different imaging modalities. In: Sharma DK Sharma R Peng S-L , editors. Proceedings of International Conference on Next-Generation Communication and Computing. Springer Nature, Singapore (2025). p. 505–19. doi: 10.1007/978-981-96-3725-6_39

[9] Link TM Kazakia G . Update on imaging-based measurement of bone mineral density and quality. Curr Rheumatol Rep. (2020) 22:13. doi: 10.1007/s11926-020-00892-w 32270332 PMC7875476

[10] Diez-Perez A Brandi ML Al-Daghri N Branco JC Bruyère O Cavalli L . Radiofrequency echographic multi-spectrometry for the in-vivo assessment of bone strength: state of the art—outcomes of an expert consensus meeting organized by the European Society for Clinical and Economic Aspects of Osteoporosis, Osteoarthritis and Musculoskeletal Diseases (ESCEO). Aging Clin Exp Res. (2019) 31:1375–89. doi: 10.1007/s40520-019-01294-4 31422565 PMC6763416

[11] Icătoiu E Vlădulescu-Trandafir A-I Groșeanu L-M Berghea F Cobilinschi C-O Potcovaru C-G . Radiofrequency echographic multi spectrometry—A novel tool in the diagnosis of osteoporosis and prediction of fragility fractures: a systematic review. Diagnostics. (2025) 15:555. doi: 10.3390/diagnostics15050555 40075802 PMC11898608

[12] Liu RY Liu E . Correlation between radiofrequency echographic multi-spectrometry (REMS) and dual-energy X-ray absorptiometry (DXA): a systematic review and meta-analysis. Bone. (2026) 206:117820. doi: 10.1016/j.bone.2026.117820 41651205

[13] Chan D Chen W Yabsley E Pocock N . Demographic determinants of REMS-derived BMD and fragility score. Osteoporos Int. (2026) 37:1125–1133. doi: 10.1007/s00198-026-07960-4 41872364 PMC13553624

[14] Bobelyak M Vaculik J Stepan JJ . Bone mineral density assessment using radiofrequency echographic multispectrometry (REMS) in patients before and after total hip replacement. Osteoporos Int. (2025) 36:2237–44. doi: 10.1007/s00198-025-07685-w 40928527 PMC12628398

[15] Belaya Z Belova K Biryukova EV Dedov II Dzeranova LK Drapkina OM . Federal clinical guidelines for diagnosis, treatment and prevention of osteoporosis. Osteopor Bone Dis. (2021) 24:4–47. doi: 10.14341/osteo12930

[16] Mazurov VI Zhugrova ES Belyaeva IB Grigoreva AL Shimanski DA Ivanov SN . Comparative study of the results of radiofrequency echographic multispectrometry and dual-energy X-ray absorptiometry in determining bone mineral density. Herald North-Western State Med Univ named after II Mechnikov. (2025) 17:62–9. doi: 10.17816/mechnikov678782

[17] Caffarelli C Al Refaie A Mondillo C Versienti A Baldassini L De Vita M . Radiofrequency echographic multispectrometry (REMS): a new option in the assessment bone status in adults with osteogenesis imperfecta. J Imaging. (2023) 9:210. doi: 10.3390/jimaging9100210 37888317 PMC10607336

[18] Adami G Arioli G Bianchi G Brandi ML Caffarelli C Cianferotti L . Radiofrequency echographic multi spectrometry for the prediction of incident fragility fractures: a 5-year follow-up study. Bone. (2020) 134:115297. doi: 10.1016/j.bone.2020.115297 32092480

[19] Ishizu H Shimizu T Sakamoto Y Toyama F Kitahara K Takayama H . Radiofrequency echographic multispectrometry (REMS) can overcome the effects of structural internal artifacts and evaluate bone fragility accurately. Calcif Tissue Int. (2024) 114:246–54. doi: 10.1007/s00223-023-01167-z 38127125

[20] Di Paola M Gatti D Viapiana O Cianferotti L Cavalli L Caffarelli C . Radiofrequency echographic multispectrometry compared with dual X-ray absorptiometry for osteoporosis diagnosis on lumbar spine and femoral neck. Osteoporos Int. (2019) 30:391–402. doi: 10.1007/s00198-018-4686-3 30178159

[21] Davidson S Vecellio A Flagstad I Holton K Bruzina A Lender P . Discrepancy between DXA and CT-based assessment of spine bone mineral density. Spine Deform. (2023) 11:677–83. doi: 10.1007/s43390-023-00646-5 36735159

[22] Sharashkina NV Naumov AV Dudinskaya EN Khovasova NO Tokareva LG Polyanskaya AR . Expert consensus: diagnosis of osteoporosis and sarcopenia in elderly and senile patients (abridged version). Therapy. (2023) 9:7–20. doi: 10.18565/therapy.2023.10.7-20

